# Tumor Necrosis Factor α Blockade: An Opportunity to Tackle Breast Cancer

**DOI:** 10.3389/fonc.2020.00584

**Published:** 2020-04-22

**Authors:** María Florencia Mercogliano, Sofía Bruni, Patricia V. Elizalde, Roxana Schillaci

**Affiliations:** ^1^Laboratorio de Biofisicoquímica de Proteínas, Instituto de Química Biológica de la Facultad de Ciencias Exactas y Naturales-Consejo Nacional de Investigaciones Científicas y Técnicas (IQUIBICEN-CONICET), Buenos Aires, Argentina; ^2^Laboratory of Molecular Mechanisms of Carcinogenesis, Instituto de Biología y Medicina Experimental (IBYME-CONICET), Buenos Aires, Argentina

**Keywords:** TNFα, breast cancer, resistance, mucin 4, targeted-therapy

## Abstract

Breast cancer is the most frequently diagnosed cancer and the principal cause of mortality by malignancy in women and represents a main problem for public health worldwide. Tumor necrosis factor α (TNFα) is a pro-inflammatory cytokine whose expression is increased in a variety of cancers. In particular, in breast cancer it correlates with augmented tumor cell proliferation, higher malignancy grade, increased occurrence of metastasis and general poor prognosis for the patient. These characteristics highlight TNFα as an attractive therapeutic target, and consequently, the study of soluble and transmembrane TNFα effects and its receptors in breast cancer is an area of active research. In this review we summarize the recent findings on TNFα participation in luminal, HER2-positive and triple negative breast cancer progression and metastasis. Also, we describe TNFα role in immune response against tumors and in chemotherapy, hormone therapy, HER2-targeted therapy and anti-immune checkpoint therapy resistance in breast cancer. Furthermore, we discuss the use of TNFα blocking strategies as potential therapies and their clinical relevance for breast cancer. These TNFα blocking agents have long been used in the clinical setting to treat inflammatory and autoimmune diseases. TNFα blockade can be achieved by monoclonal antibodies (such as infliximab, adalimumab, etc.), fusion proteins (etanercept) and dominant negative proteins (INB03). Here we address the different effects of each compound and also analyze the use of potential biomarkers in the selection of patients who would benefit from a combination of TNFα blocking agents with HER2-targeted treatments to prevent or overcome therapy resistance in breast cancer.

## Introduction

Breast cancer comprises 24.2% of total cancers and is the leading cause of cancer mortality in women worldwide (15.0%), constituting a complex problem for public health. Breast cancer is a heterogeneous disease comprised by different subtypes which have distinct characteristics, therapies, and survival rates. TNFα, on the other hand, is a pleiotropic cytokine that participates in a wide spectrum of processes with contradictory effects which range from cell survival, proliferation, immunostimulation to cell death, and immunosuppression. The final effect depends on the concentration and context in which it is found. TNFα, as a central inflammation mediator, has been linked to breast cancer initiation, progression, and metastasis [reviewed in (1)]. Given these facts, understanding TNFα's pivotal role in breast cancer is of great importance, and evidence highlights this cytokine as a novel target to develop therapies that could be combined with the current breast cancer treatments to overcome or avoid resistance and achieve a better clinical outcome, as well as to reduce the social cost of the disease.

## TNFα and its Receptors: an Overview

TNFα was named due to original research that in 1975 determined that TNFα causes hemorrhagic necrosis of tumors when found in high concentration (2–4). Later, it was discovered that TNFα was involved in a plethora of cellular processes and, more importantly, that it had paradoxical effects. It was initially described that TNFα was mainly produced by activated macrophages, monocytes, NK cells, T lymphocytes, neutrophils and mast cells, but afterwards it was discovered that it was also expressed by non-immune cells like fibroblasts, endothelial cells, cardiac myocytes and neurons, among others (5, 6). TNFα expression is induced transcriptionally by nuclear factor κB (NF-κB) (7), c-Jun, activator protein 1 (AP1) and nuclear factor associated with activated T lymphocytes (NFAT) (6).

TNFα is encoded by a single copy gene located in human chromosome 6 and is closely located to the genes of the major histocompatibility complex (8). It has four exons and three introns, but most of the protein is encoded in the last exon, while the first ones contain the leader peptide sequence. TNFα is synthesized as a type II membrane protein of 26 kDa that forms a homotrimer and is known as transmembrane TNFα (tmTNFα). Interestingly, tmTNFα can act both as a ligand by binding mainly to TNFα Receptor 2 (TNFR2) or can function as a receptor itself (9). tmTNFα on the cell surface can bind to its receptors on the target cell and modulate either physiological or pathological responses which are not only related to the immune system. For example, tmTNFα is involved in host defense (10), regulation of cytotoxic activity against cells and tumors (11), immunoglobulin production by B cells (12, 13), activation of T lymphocytes (9) and NK cells (14), stimulation of monocytes to generate cytokine production (15), activation of endothelium (9, 13), adipocyte differentiation (16) and cardiac hypertrophy (17), among others. When acting as a receptor, tmTNFα is said to “reverse signal” (18, 19), as other TNFα family members, like CD30L (20), CD40L (21), CD137L (22), meaning that it signals outside-to-inside back to the tmTNFα expressing cell (23). Although reverse signaling has been studied mostly in activation and modulation of the immune system, this mechanism has not been completely characterized (19). tmTNFα has an external C-terminus and a cytoplasmic N-terminus that is liberated by proteolytic cleavage, rendering an active soluble mature TNFα (sTNF) of 17 kDa by TNFα converting enzyme (TACE/ADAM17) (24, 25). This TNFα monomer forms homotrimers of a total molecular weight of 52 kDa with a very potent autocrine, paracrine, and endocrine effect (26). However, the sequence and mechanisms that lead this selective cleavage process remain unclear (27).

Both tmTNFα and sTNFα can bind to two membrane receptors: TNFα Receptor 1 (TNFR1/CD120a, 55 kDa) and TNFR2/CD120b (75 kDa) (28) causing their trimerization, a process required to recruit signaling proteins to its cytoplasmic domain to form an adaptor scaffold that will trigger different pathways depending on which receptor is activated, the cell type and the overall cellular context.

TNFR1 and TNFR2 are members of the TNFα receptor superfamily, which is characterized by the presence of cysteine clusters and are classified as type I membrane proteins. The extracellular ligand binding domain of each receptor shares only 28% homology, which explains the broad spectrum of processes that this superfamily can control. The intracellular domains of the receptors have no sequence homology and are void of enzymatic activity, therefore triggering signaling through recruitment of cytosolic adaptor proteins (29). To add yet another layer of complexity to the TNFα pathway, both receptors can be found in soluble forms and are proteolytically cleaved by TACE, as is the sTNFα form. These soluble receptors regulate availability of TNFα by sequestering it, or they can protect it from degradation, enabling it to achieve a sustained signal (30, 31). TNFR1 is expressed at low levels in the majority of nucleated cell types [reviewed in (32)] and can be activated by both sTNFα and tmTNFα. TNFR2 is mostly expressed in restricted cell subtypes like neurons, oligodendrocytes, astrocytes, endothelial cells, and subpopulations of T lymphocytes, among others (33, 34), and tmTNFα is required for its full activation.

Signal transduction through TNFR1 can induce opposite effects: it can either stimulate cell survival and proliferation, or apoptosis and cell death, depending on signal strength, the signaling molecules recruited to the scaffold and on the crosstalk with other pathways (35). TNFR1 has a cytoplasmic death domain that recruits TNFR1-Associated Death Domain protein (TRADD) and TNF Receptor Associated Factor 2 (TRAF2). It can form two different complexes, depending on the engaged scaffold: complex I culminates in the activation of survival pathways stimulating signaling mediated by JNK, NF-κB, AP-1, MAPK pathways (36). On the other hand, the formation of complex II recruits Fas-Associated protein with Death Domain (FADD) and pro-caspases (37) which in turn create a death-inducing signaling complex that concludes in apoptosis (38).

On the contrary, TNFR2 primarily stimulates cell activation, migration, and proliferation (39). Even though TNFR2 lacks the death domain, it can also bind TRAF2 through TNFα Receptor Associated Factor 1 (TRAF1) and can activate the canonical and non-canonical NF-κB pathway just like TNFR1, but the process is slower (40) and the activation is more sustained (41). There have been some other descriptions of TNFR2 activating TNFR1 pathways, such as NF-κB, AP-1, JNK and MAPK (34, 42), by recruiting scaffold proteins such as Receptor Interacting Protein (RIP-1) and TRADD via TRAF2, resulting in apoptosis as well as in TNFR1 activation. Given that TNFR2 has lower affinity for TNFα than TNFR1, it can augment TNFα concentration near TNFR1 and constitute the so-called ligand passing process, thereby stimulating TNFR1 signaling (43).

In summary, the intricate pathway of TNFα accounts for two receptors that can be found in a transmembrane or soluble form and can activate unique signaling cascades, but can also converge, depending on the adaptor proteins recruited. In addition, TNFα also exists in a transmembrane and soluble form, adding further layers of complexity to the TNFα pathway which explain the various and opposite cellular processes in which TNFα is involved.

## The Role of TNFα in the Normal Mammary Gland

In the normal mammary gland, TNFα elicits proliferation, morphogenetic branching, and differentiation of said tissue (44, 45). Varela and Ip reported that mammary epithelial rat cells express tmTNFα and sTNFα as well as both TNFRs, and that their expression is independently regulated during mammary gland development. TNFR1 is the receptor that mediates proliferation of normal epithelial cells, while TNFR2 regulates casein accumulation (46). TNFα effect in the normal mammary gland is predominantly exerted by NF-κB in normal rat mammary epithelial cells in culture (47). Interestingly, Varela et al. showed that p50/50 NF-κB homodimers were the ones to mediate TNFα effects, unlike the classical heterodimer of p50/p65 (47).

During the different stages of mammary gland differentiation, TNFα expression is tightly regulated and its concentration varies. Through puberty, TNFα is present to stimulate branching morphogenesis, increasing expression of MMP9 to allow tissue remodeling (45). In the course of pregnancy, TNFα stimulates proliferation and morphological differentiation but inhibits casein accumulation via TNFR1 until lactation (46). This effect is mediated by TNFα-induced NF-κB pathway activation and by TNFα repression of STAT5 phosphorylation in mammary epithelial cell lines *in vitro* (48), but it has also been reported that NF-κB could be activated by other factors such as EGFR (49). During lactation, sTNFα decreases, while tmTNFα is expressed at high levels like both TNFRs. Therefore, NF-κB pathway activation is reduced due to diminished nuclear p50 and p65 (48). Finally, during involution of the mouse mammary gland *in vivo*, sTNFα is the main form found. It acts via TNFR1, activating TWEAK (50, 51), LIF, p42/p44 MAPK and STAT3 (52) to induce apoptosis (53, 54).

## The Relevance of TNFα in Cancer

The presence of inflammatory mediators in the tumor microenvironment (TME), generated either by the tumor cells or the tumor infiltrating cells, has been widely recognized as one of the hallmarks of cancer [reviewed in (55, 56)]. There is evidence that relates pro-inflammatory cytokines to cancer as functional polymorphisms in the cytokines genes have been associated with cancer severity. In addition cell populations that secrete inflammatory mediators corresponding to either tumor cells or host immune cells, inflammatory cytokines have been detected in cancer patients and associated to poor prognosis, as reviewed by Mantovani and Balkwill (57, 58). As mentioned before, TNFα is a major mediator of inflammation (59) with ambiguous effects and has been detected in human ovarian (60–62), breast (62, 63), endometrial (62), oral (64), pancreatic (65), gastric (66), liver (67), prostate, bladder and colorectal (68) cancer as well as in lymphomas (69) and leukemias. It has been reported that knockout mice for TNFα are resistant to chemical-induced carcinogenesis (70–72). There has been quite a big controversy regarding TNFα expression as a parameter to predict clinical outcome in breast cancer patients (73–76). In this sense, recent meta-analysis showed that the TNFα−308 polymorphic site (77) but not−238 (78) associates with breast cancer patient survival. The information here presented postulates TNFα as a central player in cancer initiation, progression, invasion and metastasis, and proposes it as an attractive therapeutic target for the benefit of cancer patients.

### TNFα in Breast Cancer: Signaling, Progression, and Metastasis

Low grade inflammation has a key role in cancer pathogenesis and progression [reviewed in (56, 57, 79)]. It has been reported that TNFα treatment in certain breast cancer cell lines inhibits proliferation and induces apoptosis (80). On the other hand, it has also been shown that most breast cancer cell lines are resistant to TNFα-induced apoptosis, and that their proliferation, survival, and progression are mediated by said cytokine (81). This difference could be due to the differential expression of TNFRs, of the Bcl-2 family, of caspases activation or of ceramide expression (80).

It has been previously reported that inflammation is associated with poor prognosis and a higher recurrence risk in breast cancer patients (82, 83). TNFα mRNA and protein were present in malignant and stromal cells in biopsies of breast cancer patients, and especially in those with worse prognosis (74). Lately, several studies have shown the prometastatic role of TNFα and its participation in the epithelial-to-mesenchymal (EMT) process necessary for migration of tumor cells to establish metastasis (84). In particular, prolonged exposure to TNFα of breast cancer cell lines induces the upregulation of the transcriptional repressor Twist1 via activation of IKKβ and NF-κB and induces EMT and cancer stemness properties (85). In a study of a cohort of patients with inflammatory breast cancer it was found that there was a direct correlation between TNF-α production by peripheral blood T lymphocytes and the detection of circulating tumor cells expressing EMT markers (86).

Breast cancer diagnosis is carried out by determination by immunohistochemistry of the HER2 receptor, of hormonal receptors (estrogen and progesterone receptor, ER and PR, respectively) and of the proliferation marker Ki67. According to the expression of these receptors, breast cancer is classified into the following subtypes: luminal A or B (ER-positive, PR-positive and high or low Ki67, respectively), luminal B HER2-positive (ER-positive, PR-positive, HER2-positive), HER2-positive and triple negative breast cancer (TNBC, ER-negative, PR-negative, HER2-negative) (87).

In the following sections we will address the role of TNFα in each of these subtypes and its effect on signaling, proliferation, progression, and metastasis.

### Luminal Breast Cancer Subtype

This breast cancer subtype is characterized by the presence of ER and PR and the incidence for luminal A and luminal B subtype is 30–40 and 20–30% (88), respectively. Regarding survival rate luminal A and B has the best overall survival with 92.5% at 4-years followed by luminal B/HER2-positive with 90.3% (89). Since PR expression is driven by ER, PR is said to parallel ER expression. We will therefore focus on the relationship between ER and TNFα [reviewed in (90)].

About two thirds of breast tumors express ER enabling estrogens to drive their growth. Estrogen production takes place in ovaries, muscle, liver, breast, and adipose tissue. The main sources of plasmatic estrogens in pre-menopausal women are the ovaries, and in postmenopausal women, the sources are the adipose tissue and muscle (91). In breast cancer, estradiol is considered to have the strongest estrogenic effects and also has a higher concentration than in normal breast tissue. Indeed, the levels of estradiol in breast tumors of postmenopausal women were reported to be 10–100 times higher than its serum concentration, and values were similar to those found in pre-menopausal women (92, 93). This fact was supported by the finding that the expression level of aromatase mRNA is enhanced in breast cancer, compared with normal breast tissue. Breast tumors, therefore, proved to be autonomous in retaining a constant estradiol concentration, even against a steep plasma to tumor concentration gradient in postmenopausal patients. Briefly, estradiol can be derived from testosterone, estrone or estradiol sulfate due to the catalytic activity of aromatase, 17b-hydroxysteroid dehydrogenase (17-OHSD) or estrone sulphatase, respectively. It was demonstrated by immunohistochemistry and by *in situ* hybridization that aromatase is expressed mainly in malignant human breast epithelial cells (94).

**Figure 1:**
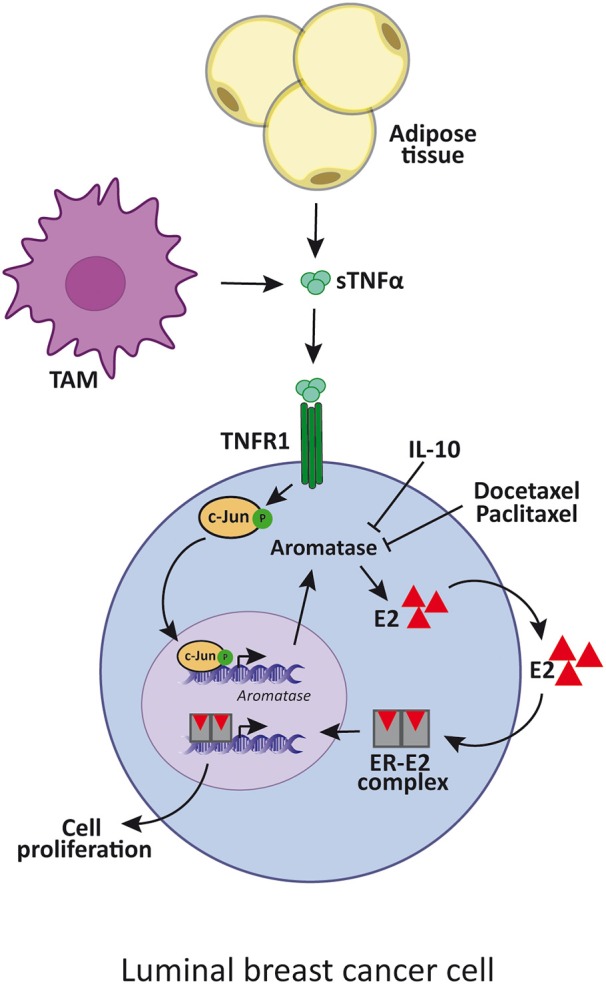
TNFα enhances luminal breast cancer cell proliferation by aromatase upregulation. TNFα is produced by adipose cells, TAM or tumor cells itself, and induces the expression of aromatase. This enzyme increases estradiol synthesis which binds to ER that, in turn, promotes luminal cancer cell proliferation. IL-10 and docetaxel and paclitaxel inhibit aromatase synthesis by reducing TNFα signaling. sTNFα, soluble TNFα; TAM, tumor-associated macrophages; E2, estradiol; ER, estrogen receptor.

Many cytokines, such as TNFα, IL-6 and PGE2, stimulate aromatase activity in primary cultured human mammary adipose tissue. In this regard, it was reported that aromatase mRNA levels positively correlate with TNFα, IL-6, and COX2 mRNA levels (95). Moreover, it was shown that TNFα induces aromatase gene expression through c-fos and c-jun binding on the AP-1 element present on exon 1.4 together with the glucocorticoid receptor (91). Considering that aromatase is only expressed in undifferentiated adipose fibroblasts but not in the mature adipocytes, it is also possible that TNFα and IL-6 contribute to augment aromatase mRNA expression by increasing this population in breast cancer, also given that both cytokines are inhibitors of adipogenic differentiation (96). On the other hand, IL-10 through inhibition of TNFα-induced p42/p44 MAPK activation can suppress aromatase mRNA expression in human adipose tissue (97) (Figure 1).

Reports in favor of the anti-proliferative and apoptotic effect of TNFα on luminal breast cancer have only been executed on the MCF-7 cell line. However, controversial results have been found since a study showed that MCF-7 lines from different laboratories had different expression levels of the anti-apoptotic protein Bcl-2, which consequently modified the sensitivity of the cells to TNFα-induced apoptosis (80).

For instance, it was reported that TNFα induces a cytotoxic effect in luminal breast cancer cell lines in absence of ubiquitin editing enzyme TNFα-induced protein 3 (TNFAIP3 also called A20) (98), but this protein has a wide range of effects in different tissues (99, 100). Not only does A20 protects cells from TNFα cytotoxic effects but it also contributes to a more aggressive phenotype in response to TNFα stimulation.

**Figure 2:**
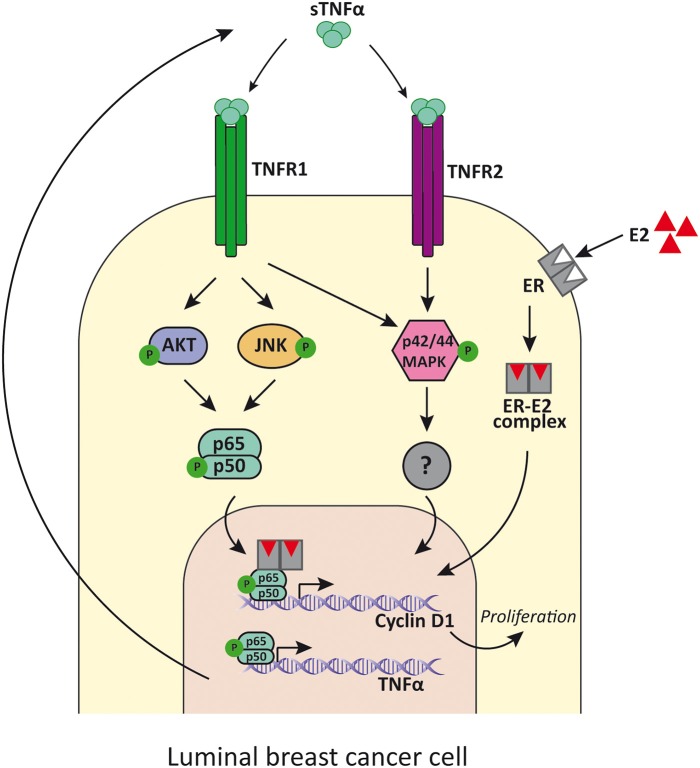
TNFα signaling in luminal breast cancer cells. TNFα binds to TNFR1 and induces the activation of PI3K/Akt, p42/p44MAPK and JNK, while its binding to TNFR2 mainly induces p42/p44MAPK activation. PI3K/Akt and JNK then activates NF-κB that promotes the transcription of cyclin D1 and the consequent cell proliferation. In addition, NF-κB also induces the expression of TNFα, establishing a positive feed-back. E2 binds to ER and also promotes luminal cancer cell proliferation through cyclin D1 upregulation by tethering NF-κB and enhances its activity. sTNFα, soluble TNFα; TNFR1, TNFα receptor 1; TNFR2, TNFα receptor 2; E2, estradiol; ER, estrogen receptor.

There have been various reports of NF-κB repression by ER accounted for different mechanisms (101), such as prevention of NF-κB binding to DNA (102), recruitment of co-repressors (103), competition for co-activators (104), and prevention of NF-κB translocation to the nucleus (105), among others. Even though clinical data reported that ER-positive breast tumors with constitutively active NF-κB are more aggressive and less responsive to treatment (106), very few studies indicated that a positive transcriptional crosstalk could exist (107, 108). It was Frasor et al. who showed that treatment with TNFα and estradiol regulated a set of genes that are clinically relevant because they can distinguish patients with poor response to endocrine treatment (109). In fact, both molecules act together to promote survival of breast cancer cells and progression onto a more aggressive phenotype (110). In this regard, by using global run-on coupled with deep sequencing (GRO-seq) in MCF-7 breast cancer cells, it was demonstrated that TNFα was responsible for exposing latent estrogen receptor binding sites to which estradiol could bind to regulate gene expression. The availability of these enhancers was dependent on TNFα induction of the NF-κB pathway and the pioneer factor FOXA1. The proliferative effects of TNFα in human breast cancer cell lines *in vitro* were shown to be mediated by TNFR1, which activates JNK and PI3K/AKT which stimulates NF-κB. The latter, in turn, induces cyclin D1 expression which concludes in cellular proliferation (Figure 2) (108, 111). Interestingly the same effects were reported for estradiol treatment (108). TNFα can also stimulate cancer cells proliferation through the p42/p44 MAPK pathway mediated by TNFR1 and TNFR2 independently of NF-κB, establishing an alternative pathway to breast cancer proliferation (111) (Figure 2). MCF-7 cell line is sensitive to TNFα-induced apoptosis through JNK pathway activation, while p42/p44 MAPK is weakly activated (112). Instead, in T-47D cell line, TNFα has proliferative effects and causes potent activation of p42/p44 MAPK, while JNK is only transiently activated (111). Moreover, when NF-κB pathway activation is insufficient, JNK stimulates apoptosis (36, 113).

Additionally, TNFα elicited metastatic properties in the originally non-metastatic MCF-7 cell line, such as cell spreading and formation of FAK/paxillin protrusions through Src activation, elevated expression of CD44, VLA6 and β1, cell migration and initiation of the EMT process, resistance to chemotherapy and secretion of pro-tumoral factors such as chemokines and cytokines (114, 115). Moreover, TNFα in combination with estradiol and EGF had a stronger effect than each molecule alone (114). EMT has been found to be accountable for the change to a fibroblast-like morphology, loss of adhesion and junction molecules, and greater invasion and cell motility (116). EMT has also been associated with the development and maintenance of breast cancer stem cells. All the above-mentioned characteristics have been linked not only to maintaining the tumor but also to the acquisition of metastatic potential, therapy resistance, and relapse (117). All these effects were proven to be exerted by TNFα in MCF-7 and T-47D cell lines (118, 119). TNFα also modulates MMPs expression, in particular MMP9, in MCF-7 cells (120). This protein family is involved in cell migration and invasion of the extracellular matrix (120, 121). All these facts show that TNFα plays a critical role in luminal breast cancer, inducing tumor promotion, proliferation, survival, progression and metastasis, and that it can be an attractive target for potential new therapies to enhance patients' successful outcome.

### TNBC Subtype

As stated before, TNBC is characterized by not expressing clinically significant levels of ER, PR and HER2 receptors. This breast cancer subtype accounts for 15–20% of breast cancers and is associated with younger women (<35 years) (122) in Hispanic and/or African American populations (123). It is usually diagnosed in an advanced stage, has a high risk of metastasis development and exhibits poor response to therapy (124, 125). Consequently, presence of TNBC has a survival rate of 77%, and together with the HER2-positive subtype are the ones that have shorter 4-year survival (89).

Regarding TNFα, there have been paradoxical results of its implication in TNBC proliferation and survival. It has been stated that TNFα can induce apoptosis (126) and promote tumor growth in TNBC, depending on the specific cell line and the cellular context in which it is found (127–129). Qiao et al. demonstrated that TNFα induces apoptosis in BT549 cells, activating c-Jun which, in turn, downregulates anti-apoptotic genes, and upregulates pro-apoptotic cascades. In the same study, they showed that TNFα can also have a pro-malignant effect, stimulating c-Jun and inducing cell invasion (126). Other groups have proved that treatment with exogenous TNFα cannot trigger apoptosis in MDA-MB-468 (127) and MDA-MB-231 cell lines (130). On the contrary, when TNFα was silenced, cell proliferation and motility were abolished and apoptosis was induced (128).

When promoting tumor growth, TNFα activates NF-κB and p38/MAPK pathways which stimulate signal transducer and activator of transcription 3 (STAT3) (127), a known transcription factor classified as an oncogene (131). This cascade generates a positive feedback loop because activated STAT3 increases the expression of HBXIP (Hepatitis B Virus X-Interacting Protein) which augments TNFR1 that will bind TNFα and continue the activation of NF-κB and p38/MAPK. MUC4, a membrane glycoprotein, has also been shown to promote cell proliferation mediated by β-catenin and an increase in cyclin D1 expression in TNBC cell lines (132).

**Figure 3:**
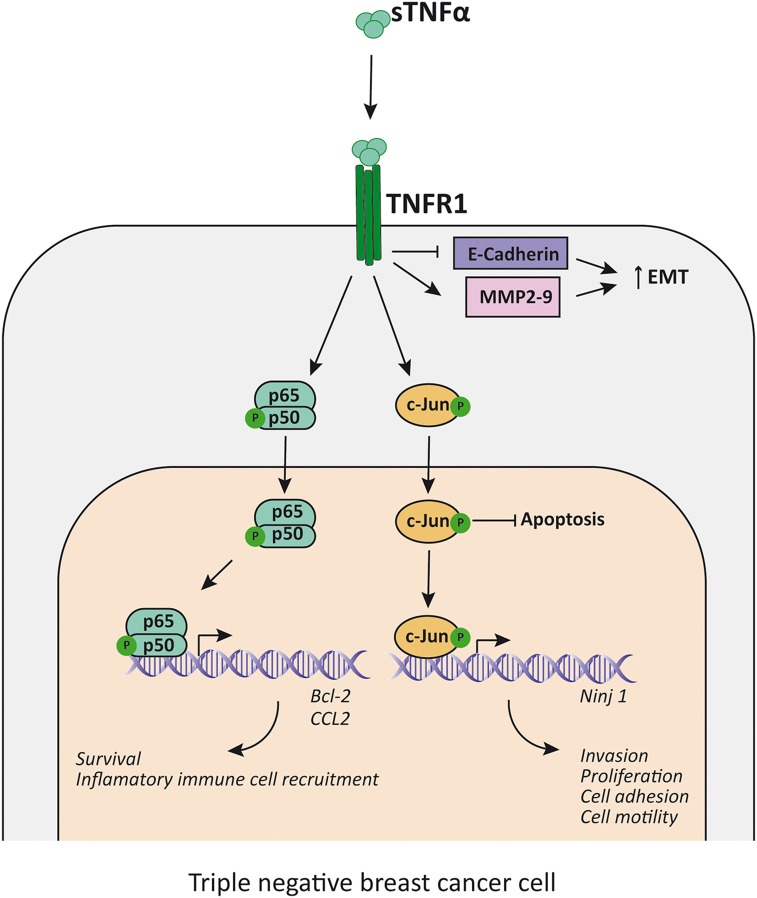
TNFα promotes proliferation and EMT in TNBC cells. TNFα, acting mainly through TNFR1, induces NF-κB and c-Jun activation, which inhibits apoptosis and enhance the transcription of survival factors (i.e., Bcl-2), the expression of inflammatory cell recruitment chemokines (i.e., CCL2) and promotes transcription of factors related to EMT (i.e., increases Ninj1, MMP2, and MMP9 transcription and decreases E-cadherin transcription). sTNFα, soluble TNFα; TNFR1, TNFα receptor 1; MMP, metalloproteinase; EMT, epithelial-mesenchymal transition.

As stated before, TNFα has been associated with cancer cell motility, invasion and EMT. TNBC is not an exception since there have been several reports of different proteins that can mediate said processes. Important players related to growth, migration and invasion in breast cancer are the MMPs (120, 121), in particular the expression of MMP9 exerted by TNFα-induced AP-1 activation (133) or CDKNA1/p21(134), which can act as an oncogene (135). It has been described that p42/p44MAPK (136) and PI3K/Akt (135) are critical for TNFα-induced MMP9 expression in cancer. Using specific pathway inhibitors, it was clear in the MDA-MB-231 cell line that the p42/p44MAPK pathway was the one responsible for TNFα-induced MMP9 expression. TNFα activates AP-1 through PI3K/Akt and p42/p44 MAPK pathways and elicits the expression of ZEB2, an EMT regulator in TNBC cell lines (137). In this regard, it has been found that PI3K/Akt and p42/p44MAPK pathways are enhanced in human TNBC samples and have a central role in EMT promotion and cell invasion in breast tumors (138) as well as in cancers in general (Figure 3).

Overexpression of A20 protein, mentioned in the luminal subtype section, is also involved in the aggressiveness of TNBC and is effectively highly expressed in this subtype according to TCGA data analysis (100). It has been suggested that A20 may induce expression of HSP70, which protects TNBC cells from TNFα cytotoxicity *in vivo* in mice models (139). It has also been demonstrated that A20 protein activates the inflammatory IL-6/phospho-STAT3 pathway, downregulates SOC3 and upregulates HSP70 and the expression of inflammatory cytokines like IL-6, IL-8, CCL5, and TGF-β upon TNFα stimulus. These events conclude in the expression of EMT markers and contribute to a more aggressive phenotype, a higher metastatic potential and the generation of cancer stem cells in TNBC cell lines (99).

We previously discussed the role of MUC4 in TNBC proliferation, but it was also found to have a pivotal position in invasion and metastasis. In this sense, MUC4 (132) and FAK (140) are overexpressed in invasive TNBC but not in normal breast tissues. MUC4 not only influences proliferation but also regulates the metastatic potential of TNBC suppressing F-actin formation and regulating FAK phosphorylation, thus increasing cell motility *in vitro* (141).

### HER2-Positive Subtype

Expression of the HER2 receptor is found in 13–20% of breast cancers (142), contributing to oncogenic transformation, a more aggressive phenotype and the worst 4-year survival (82.7%) together with the TNBC subtype (89), as already stated.

**Figure 4:**
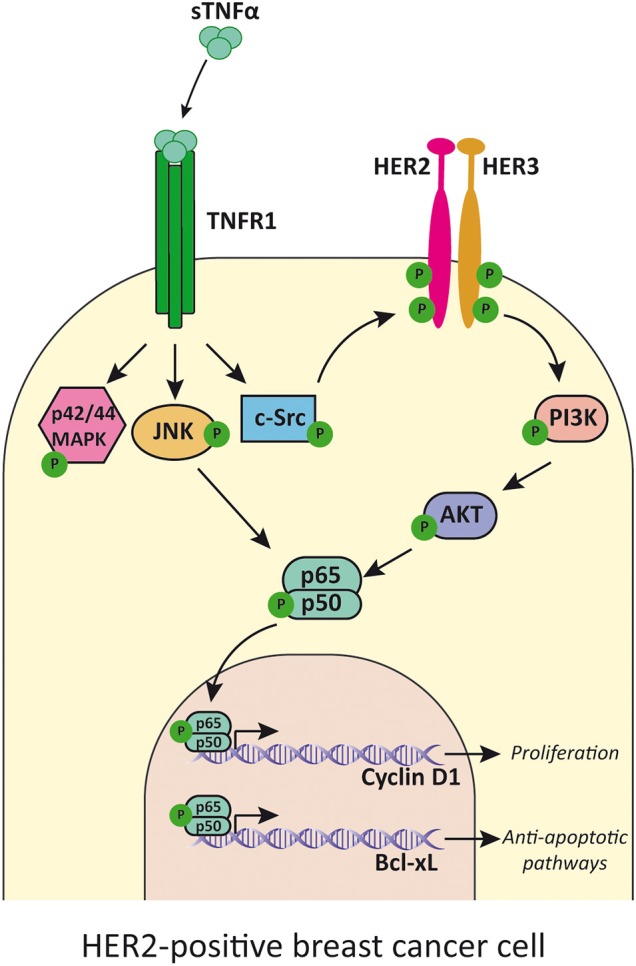
TNFα transactivates HER2 receptor and induces cell proliferation. TNFα, acting mainly through TNFR1, activates p42/p44MAPK, JNK, and c-Src. This last kinase phosphorylates HER2 and induces HER2/HER3 heterodimer formation and activation of PI3K/Akt pathway. JNK and Akt activates NF-κB that induces transcription of cyclin D1 and Bcl-xL. TNFα, soluble TNFα; TNFR1, TNFα receptor 1.

HER2 is an orphan tyrosine kinase receptor located in the cytoplasmic membrane with no known ligand, that belongs to the HER family (143). The three other members of the HER family are the EGFR/HER1, HER3, and HER4 [reviewed in (144)]. In presence of their specific ligands, the receptors can homo- or heterodimerize, upon which transphosphorylation of tyrosine residues occurs [reviewed in (145)]. This leads to recruitment of adaptor proteins or enzymes capable of activating different signaling cascades that conclude in tumor cell proliferation and survival [reviewed in (146)]. HER2/HER3 heterodimer is the duo with higher oncogenic activity (147). It stimulates primarily the PI3K/Akt and p42/p44MAPK pathways (148), but it can also activate NF-κB anti-apoptotic signaling (149). We have previously reported that in HER2-positive breast cancer cell lines, TNFα transactivates HER2, inducing phosphorylation of Tyr877 through c-Src, in a PKC-dependent manner. This elicits autophosphorylation and leads to the formation of the HER2/HER3 heterodimer which in turn activates the PI3K/Akt pathway and concludes in proliferation mediated by NF-κB-induced cyclin D1 expression (150) (Figure 4). Our group demonstrated that stable overexpression of TNFα in BT-474 cells, a human HER2-positive breast cancer cell line, led to high activation levels of Akt and NF-κB pathways (151). This overactivated signaling conferred higher survival capacity, a major colony forming potential and an increased proliferation in strict culture conditions with respect to the parental cell line (151). All these events are of major significance in the processes of tumor establishment and of an increased metastatic potential. We demonstrated that TNFα has a central role in mediating proliferative effects and tumor progression in HER2-positive breast cancer *in vitro* and in human xenograft models (150, 151).

The mucin family of proteins has been shown to be another important player in HER2-positive breast cancer. Li et al. described that heregulin stimulation, the preferred HER3 ligand, induces phosphorylation of mucin 1 (MUC1) which interacts with γ-catenin and p120 catenin (152). This complex is then translocated to the nucleus where it interacts with different transcription factors like STAT1, ERα, β-catenin, and p53 to regulate transcription of genes involved in tumor promotion (153). MUC4, another mucin family member, has been demonstrated to interact with HER2 and to promote HER2 and HER3 translocation to the cytoplasmic membrane, augmenting the number of receptors available on the cell surface and maintaining them anchored to the membrane during longer periods of time (154). Consequently, there is an increase in the signaling cascade of the HER2/HER3 heterodimer through activation of PI3K (155), causing an increase in cell proliferation and survival in HER2-positive breast cancer (156). MUC4 is aberrantly expressed in a variety of cancers, including breast tumors, and in this setting it can also induce phosphorylation of HER2, which results in inactivation of the pro-apoptotic pathway and in stimulation of pro-survival proteins (157). To our knowledge, we are the first group to report that TNFα increases MUC4 expression through activation of the NF-κB pathway in HER2-positive breast cancer (151).

## TNFα and the Immune Response Against Cancer

TNFα is known to play a dual role in oncoimmunology [reviewed in (158)] as it can behave as an immunostimulant or immunosuppresor cytokine. TNFα is crucial not only to mediate killing of several tumor types, but also for proper proliferation of T lymphocytes, B cells, NK cells, macrophages, and dendritic cells (DC). In spite of this, evidence throughout time has shown that TNFα has a critical role in the inflammatory environment that favors cancer development, acting as a tumor-promoting factor [reviewed in (159)].

Regarding its role as an immunosuppressive cytokine, emerging evidence indicates that, in multiple cancer types, TNFα stimulates and favors not only the accumulation but also the activity of certain immune cell populations, like regulatory T lymphocytes (Tregs) (160), regulatory B lymphocytes (Bregs) (161) and myeloid-derived suppressor cells (MDSCs) (162), in different cancer types. All of the previous mentioned cell types are crucial negative modulators of tumor immune surveillance. A recent study has demonstrated that inflammatory molecules such as PGE2 (163) interleukin-1β (IL-1β) (164) and IL-6 (165) produced by tumor cells induce the stem cells present in the bone marrow of tumor-bearing mice to differentiate into Gr-1+CD11b+ MDSCs and to accumulate in the tumor tissue. This, in turn, allows tumor progression as it impairs the activation of T lymphocytes due to the already known potential of this cell population to suppress not only the innate but also the adaptive immune response (166). In addition, the accumulation of MDSCs in the tumor bed of ovarian cancer was shown to be regulated by the production of CXCL12 and CXCR4, induced by PGE2 (167). In line with this, it has been proved that TNFα is able to affect the leukocyte extravasation by regulating chemokine expression (168) [reviewed in (169)]. For example, in ovarian cancer cell lines, TNFα can behave as a signaling molecule within the producing cell or among the surrounding ones, and can induce CXCR4 expression through NF-κB, enhancing in this way cell invasion (170, 171). Moreover, it has been proved that TNFα secretion by MDSCs in the tumor bed acted as an autocrine cytokine that enhances their T lymphocyte suppressive activity by upregulating different genes associated with immunosuppression, like *Nos2* (172).

With respect to the role of TNFα and its signaling pathways in regulating the immune system in cancer, it has been reported that the TNFR2-mediated signaling has a critical role not only in the survival (162) but also in the activity (173) of the MDSCs. In line with this, it has been recently demonstrated that deficiency in either of the two TNFRs caused a diminished accumulation of MDSCs in the tumor bed and the cause underlying this effect was the downregulation of CXCR4 expression on the surface of MDCSs in a breast cancer model (166). This supports the idea that TNFα is involved in differentiation, migration, activation, and survival of the MDSCs in the TME. As mentioned above, TNFR1 has been reported to be the main mediator of TNFα-induced apoptosis (36). However, it was not the scenario that emerged in the majority of cancer cells, because they lost sensitivity to TNFα secreted by CD8+ T lymphocytes and NK cells in melanoma (174). There are several mechanisms involved in the resistance to TNFα-induced cell death that include the relatively high proportion of TNFα receptors associated with anti-apoptotic proteins, such as cFLIP, cIAPs, TRAF2, and the loss of caspase-8 and of 2-aminoethanethiol dioxygenase (*Ado*), a metabolic protein required for TNFα cytotoxicity (175) [reviewed in (176–178)]. Supporting this evidence, it has been proved that TNFR1 gene-deficient mice showed reduced chemical-induced colon inflammation and tumor incidence (179), indicating a putative role of TNFR1 in inflammation and tumor progression. In addition, posttranslational modifications of TNFR1 are known to affect its activity. It was shown that addition of α2-6 sialic acid to TNFR1 by the Golgi enzyme ST6Gal-l sialyltransferase blocks apoptosis induced by TNFα in human monocytic cells as well as in monocytes from mice that overexpressed the enzyme (180). In a recent study, Holdbrooks et al. showed that through this sialylation-dependent mechanism, TNFR1 internalization induced by TNFα was inhibited. Interestingly, this process led to the blockade of the apoptotic pathway guided by TNFR1 signaling, promoting sustained activation of the NFκB- and Akt-mediated survival pathway (181). These results indicate that in aberrant glycosylation conditions, TNFR1 can switch from the classical apoptotic signaling pathway activated by sTNFα to the one commonly attributed to TNFR2, which favors tumor progression by promoting cell proliferation.

As stated before, TNFR2 has been related to immunosuppression, tumor progression, and metastasis. Studies demonstrated that development and expansion of Treg cells in the thymus is facilitated by TNFR2 (182, 183) and that it mediates the stimulatory effects of TNFα on Treg cells (184, 185). Chen et al. proved that no other T lymphocyte expressed higher levels of TNFR2 than Treg cells, which correlated with a more suppressive population (186). Generally, the TNFα-TNFR2 axis plays a very important role in the enhancement of tumor immune escape (173). In addition, recent preclinical studies showed that TNFR2 blockade was enough to reduce the progression of breast cancer cell lines treated with TNFα (111) and that it promoted TNFα-associated lung cancer cell death (187). Nie et al. obtained inhibition of breast and colon cancer progression by combining TNFR2-blocking antibody with an anti-CD25 antibody (188).

Studies show that TNFα promotes activation-induced cell death (AICD) of CD8+ T lymphocytes (189) through TNFR2 signaling and that it prevents them from infiltrating the tumor bed. TNFR2 expression can be upregulated on CD8+ T lymphocytes (190) and can induce the expression of the inhibitory receptor Tim3 (191). Another evidence of the ability of TNFα to impair the function of antitumor immune cells is that, in melanoma, the cytotoxic response against the tumor that is carried out by CD8+ T lymphocyte is inhibited by this cytokine, which is produced by CD4+ lymphocytes infiltrating the tumor (192). In addition, in an adoptive CD8+ T cell transfer protocol, TNFα induced the dedifferentiation of melanoma cells, facilitating immune escape and melanoma relapse (193). More recently, TNFα has been shown to promote the expression of PD-L1 in cancer cells (194), leading to immunosuppression. Torrey et al. proved that targeting TNFR2 with antagonistic antibodies inhibits not only proliferation of cancer cells but also proliferation of Tregs infiltrating the tumor while simultaneously inducing the expansion of effector T lymphocytes (195).

It has been shown that the antitumor immunity promoted by the crosstalk between DC and NK cells both in human and mouse cancer models is a result of tmTNFα-TNFR2 interaction that triggers the secretion of Th1 cytokines (14, 196) [reviewed in (197)]. Impairing sTNFα-TNFR1 interaction by different strategies (sTNFα blockade, gene deletion or antibody blockade of TNFR1) can prevent autoimmunity, shedding light on the specific role of the sTNF-TNFR1 axis in inflammation (198) [reviewed in (199)]. sTNFα has been associated with all stages of cancer progression, including tumorigenesis, proliferation, angiogenesis, metastasis, and immune escape [reviewed in (158)].

Sobo-Vujanovic et al. have shown that sTNFα blockade with a dominant negative protein specific for sTNFα, INB03, or TNFR1 deletion not only prevented carcinogenesis but also decreased tumor growth and increased survival of methylcholanthrene-injected mice (71). Moreover, they proposed a new role for sTNFα during carcinogenesis in driving MDSCs accumulation in the TME, in acquiring and maintaining their immunosuppressive functions (200), in survival extension (162), and growth stimulated by IL1α, VEGF and GM-CSF [reviewed in (201, 202)]. The authors suggested that this occurred by activation of TNFR1 signaling on MDSCs progenitors, leading to MDSCs generation but also to arrested differentiation (71).

**Figure 5:**
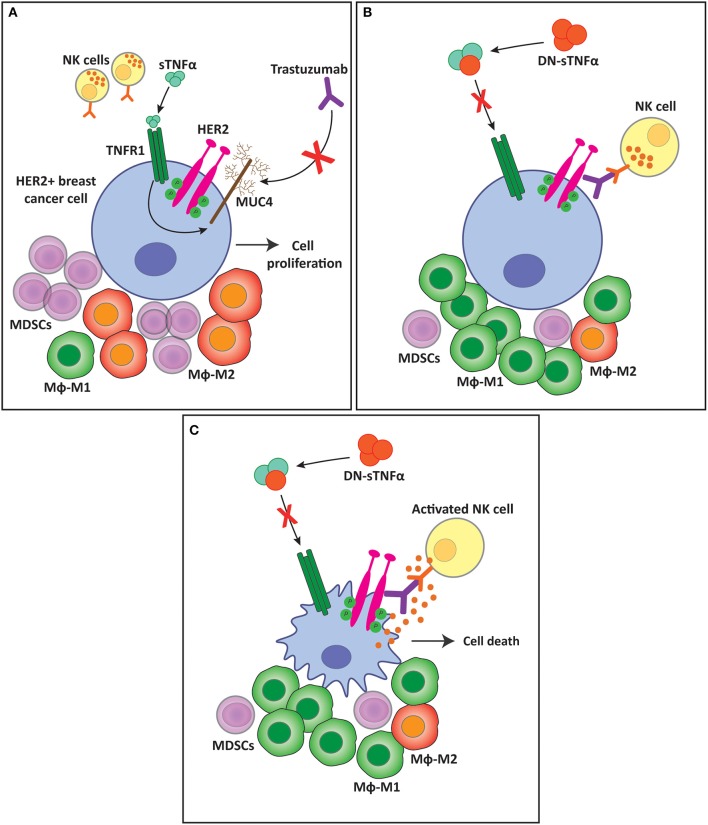
sTNFα blockade overcomes trastuzumab resistance and favors a less immunosuppressive tumor microenvironment. **(A)** sTNFα induces the upregulation of the membrane glycoprotein MUC4 in HER2-positive breast tumors. MUC4 masks trastuzumab epitope on the HER2 molecule, impairing its binding and ADCC exerted by NK cells, generating resistance to the antibody. This resistance is accompanied by an immunosuppressive tumor microenvironment with an increased infiltration of MDSCs and macrophage polarization to the M2 subtype. **(B)** sTNFα blockade with a dominant negative protein (DN-sTNFα) downregulates MUC4 expression, enabling trastuzumab to induce NK cell ADCC. This antitumor innate immune response generates a less immunosuppressive tumor microenvironment, decreasing MDSCs infiltration, and increasing macrophage polarization to the M1 subtype. **(C)** NK cell activation and degranulation induced by trastuzumab treatment kills tumor cells through ADCC. sTNFα, soluble TNFα; TNFR1, TNFα receptor 1; MUC4, mucin 4; MDSC, myeloid-derived suppressor cells; Mϕ, macrophages.

Results from our own group support the idea that TNFα is engaged in mounting an immunosuppressive TME during carcinogenesis, particularly through the sTNFα isoform. In a suitable breast cancer preclinical model, we demonstrated that HER2-positive tumors that are *de novo* resistant to trastuzumab, an anti-HER2 monoclonal antibody used as a first line treatment for patients with this type of cancer, are sensitized when sTNFα is blocked with INB03 in combination with trastuzumab (203) (Figure 5). Together with tumor growth inhibition, we found that sTNFα blockade with INB03 in combination with trastuzumab increases tumor infiltrating NK cell activation and degranulation, increases macrophage recruitment to the tumor bed, and favors their differentiation to the M1 subtype. In addition, these tumors exhibited a decrease in myeloid cell infiltration and in the percentage of the monocytic subtype of the MDSCs population (203) (Figure 5). These results indicate that sTNFα is responsible for generating an immunosuppressive TME and favoring tumor immune evasion.

In line with our results, a recent study has demonstrated that HER2-positive breast cancer patients exhibited a specific tumor transcriptome that conditioned their response to adjuvant trastuzumab (204). The study shows that HER2-positive tumors that were sensitive to trastuzumab treatment had a higher expression of certain chemokines related to an antitumor immune response, to migration of T and B lymphocytes to the tumor bed and monocyte recruitment. In particular, patients bearing trastuzumab sensitive tumors showed an upregulation of CCL2 expression in contrast to the HER2-positive tumors that were resistant. The expression profile of the tumors that responded to therapy correlated with an increased CD8+ T lymphocyte infiltration of the tumor and an enrichment of the macrophage M1 subtype genes, suggesting macrophage polarization. In particular, it is proposed that CCL2 recruits mainly monocytes to the TME and that its chemokine action is regulated by the PI3K/NF-κB pathway, activated by the HER2 receptor in the plasma membrane. Triulzi et al. claim that there is a strong relationship between the expression of immune chemokines like CCL2 together with the infiltration of immune cells in the TME and with the efficacy of trastuzumab treatment in HER2-positive patients (204).

## Involvement of TNFα and Nf-κB in Breast Cancer Therapy Resistance

Breast cancer treatment with radiotherapy, endocrine treatment, chemotherapy, targeted therapies or immune checkpoint drugs induces, in the majority of cases, an excellent initial tumor response. However, survival of cancer cells after the first line of treatment induces cancer recurrence or their lack of response leads to resistance events that are life-threatening. As described previously in this review, TNFα is not only closely involved in breast cancer onset, progression and in metastasis formation, but it also participates by favoring therapy resistance. As NF-κB is one of the main transcription factors activated by TNFα, we will here also highlight the primary data pointing out to the relationship between NF-κB and resistance to breast cancer therapies. Table 1 shows the reported mechanisms by which TNFα induces resistance to chemotherapy, radiotherapy, tyrosine kinase inhibitors, PARP inhibitors, trastuzumab and anti-immune checkpoint therapies in breast cancer.

**Table 1:** Mechanisms of TNFα-induced therapy resistance in breast cancer.

**Breast cancer treatment**	**Drug**	**Mechanism**	**Experimental setting**	**References**
Endocrine therapy	Tamoxifen	ER phosphorylation at Ser118	MCF-7 cells	(205, 206)
	Fulvestrant	ER phosphorylation at Ser118	MCF-7 cells	(206)
Chemotherapy	Mitoxantrone	ABCG2 transporter	MCF-7 cells	(207)
	Doxorubicin	GST-π	MCF-7 and MDA-MB-231 and human breast cancer samples	(208)
	Docetaxel	Reduced levels of TNFR1	MCF-7 MDA-MB-231and A2780 cells	(209)
Radiotherapy	Ionizing radiation	NF-κB activation	UACC-893, HCC70, and BT474 cells	(210)
			MCF-7 cells	(211)
Tyrosine-kinase inhibitor	Lapatinib	NIBP/IKKß/NF-κB activation	BT474, SKBR3 and ZR75.30, HCC202, VP229, HCC1569, MDAMB453, HCC1954, JIMT1, and MDAMB361 cells	(212)
		Ca2+ signaling	BT474 and SKBR3	(213)
	Erlotinib^*^	TRAF2-RIP1-IKK	11–18 tumor xenografts, HCC827 EGFR exon19 deletion, 11-18 EGFR L858R, H3255 EGFR L858R, PC9 exon19 deletion, and H1975 EGFR L858R/T790M	(214)
		miR21 decrease, TNFα production	18 NSCLC cell lines, xenograft EGFRmutant HCC827 and EGFRwt NSCLC A549 cells and in an EGFRwt patient-derived xenograft (PDX) model (HCC4087), human NSCLC samples	(215)
	Pictilisib	Macrophage-induced NF-κB activation	4T1 cells *in vitro* e *in vivo*	(216)
PARP inhibitor	Olaparib	NF-κB activation	UWB1.289 ovarian HCC1937 breast cancer cells	(217)
Anti-HER2 monoclonal antibody	Trastuzumab	MUC4 expression	BT-474, SK-BR3, JIMT-1, KPL-4 and NCI-N-87 cells, *in vitro*; KPL-4, and JIMT-1 xenografts; breast cancer samples	(150)
Immune checkpoint blockade	anti-PD-L1	PD-L1 mRNA estabilization	BT549, 4T1, MB231, and MB468 cell *in vitro*; 4T1 cells and EMT6 *in vivo*	(198)
	anti-PD-1	TIM-3 expression	B16K1 melanoma, Lewis lung carcinoma (LLC) and 4T1 breast cancer cells *in vitro* and in vivo	(195)
		TRAF2	BLM, SK-MEL-147, D10, SK-MEL-23, SK-MEL-28, A375, 888-mel, A875, HEK293T melanoma cells, HCC827, LCLC-103H and HCC4006 lung cancer cells *in vitro*; M032.X2.CL and M026.X1.CL cells xenografts	(181)

* *Proved in lung cancer*.

### Chemotherapy and Radiotherapy

Radiation therapy or chemotherapy along with radiation treatment combined with breast-conserving surgery (or mastectomy), are the therapeutic approaches used for all breast cancers subtypes. Although these treatments provide a striking increase in survival, tumor recurrence due to chemotherapy and radiotherapy resistance remains a major clinical problem to the successful treatment of breast cancer.

One of the known mechanisms of chemotherapy resistance relies on the ATP binding cassette transporter G2 (ABCG2, also known as breast cancer resistance protein), that causes efflux of several endogenous and exogenous agents, such as chemotherapy drugs, thus conferring a drug resistant phenotype [reviewed in (218)]. In particular, ABCG2 is able to exclude drugs such as mitoxantrone, captothecin, indolocarbazole, topoisomerase I inhibitors, EGFR1 inhibitors, methotrexate, flavopiridol, and quinazoline from the cells (219). Pradhan et al. demonstrated that TNFα synergized with the estradiol effect on ABCG2 expression. This effect was mediated by exposure of a latent NF-κB response element in the ABCG2 promoter induced by estradiol treatment, which allowed NF-κB binding near ER binding site and led to an increase in ABCG2 mRNA and protein expression (220). In the MCF-7 cell line resistant to mitoxantrone, a type II topoisomerase inhibitor which disrupts DNA synthesis, TNFα induced an increase in ABCG2 protein levels (221). These findings are in line with a previous report exploring the positive crosstalk between ER and NF-κB that revealed ABCG2 as a common target of both transcription factors that induced aggressiveness of breast cancer (109). Doxorubicin treatment of chemotherapy-resistant cell lines, such as T-47D and MBCDF, induced NF-κB activation which persisted longer in time than in the sensitive cell lines. This effect was due to a non-canonical phosphorylation of IκBα through c-Abl kinase (222).

Gemcitabine, a nucleoside analog that acts as an antimetabolite by arresting cells in the S-phase of cell cycle, has been widely used in the treatment of several solid tumors, including breast cancer (207). It was demonstrated in MDA-MB-231 cells that gemcitabine treatment increased TNFα production and the transcriptional activation of NF-κB through IκBα phosphorylation. Phosphorylation blockade with BAY11-7082 increased the cytotoxic activity of gemcitabine, suggesting that NF-κB is promoting breast cancer cell survival in this model. A similar behavior was obtained in MCF-7 cells treated with docetaxel. TNFα levels increased upon docetaxel treatment and were associated with the induction of docetaxel resistance, cell survival dependent on TNFR2/NF-κB signaling, and degradation of TNFR1. tmTNFα has also been linked to chemotherapy resistance. In this case, tmTNFα expressed in breast cancer cells, transmit outside-to-inside signals in the cells promoting activation of NF-κB through the p42/p44 MAPK signaling pathway to promote glutathione S-transferase- π (GST-π) expression, an enzyme that reduces intracellular doxorubicin levels. In addition, inhibition of tmTNFα expression improved sensitivity to docetaxel in breast cancer cells *in vivo* and *in vitro*. In breast cancer patient samples, tmTNFα expression was associated with tumor size, incidence of metastasis, and HER2 expression, while it was absent in peritumoral breast tissue (223).

Radiotherapy is frequently applied as a standard protocol after surgery. There are reports in chemotherapy resistance that indicate that NF-κB is highly activated in irradiated cells. The irradiation of MCF-7 cells with low dose of ionizing radiation (2 or 10 Gy of 137Cs γ-rays) proved to induce two phases of NF-κB activation (209). The first was triggered by the irradiation itself and induced an increase in TNFα transcription which led to a second phase of sustained NF-κB activation. TNFα from irradiated cells activated NF-κB in the non-targeted unirradiated bystander cells as well, establishing a feedforward effect. This data suggested that the TNFα/NF-κB pathway induced by irradiated cells could support the survival and progression of tumor cells. In addition, TNFα pretreatment of breast cancer cells protected them from radiation insults (208, 211).

In the clinical setting, the evaluation of NF-κB in pre- and post-chemotherapy breast cancer samples from patients subjected to neoadjuvant chemotherapy with anthracycline- and/or taxane, showed that activation of NF-κB was associated with chemoresistance. Furthermore, nuclear NF-κB staining was more frequently present in breast cancer cells after chemotherapy (210). As reported for endocrine-resistant breast cancer, NF-κB activation promoted the expression of the inhibitors of apoptosis (IAPs) and Bcl-xL, contributing to a chemotherapy and radiotherapy resistant phenotype which was also associated with treatment response [reviewed in (224, 225)].

### Endocrine Therapy

As stated previously, the proliferative effects of estradiol in breast cancer are mediated by ER. Several therapeutic strategies were developed to target this axis. Tamoxifen is a pioneer selective estrogen receptor modulator that competitively inhibits estrogen binding to ER in breast tissue and has been widely used to treat ER-positive breast cancers in pre- and post-menopausal women (226). Fulvestrant is a pure antagonist that blocks ER, induces its downregulation, and promotes interactions with corepressors (227). Another option to tackle the estradiol/ER pathway is to inhibit estradiol production by aromatase inhibitors. Currently, the third generation of aromatase inhibitors, such as anastrozole and letrozole, is used in post-menopausal women with breast cancer (228). Despite these therapies, over 30% of patients with ER-positive breast cancer experience relapse, a fact that highlights the study of the resistance mechanisms to tamoxifen, fulvestrant and aromatase inhibitors, as an important field of research [reviewed in (229)]. With reference to TNFα participation in hormone therapy resistance, Castellaro et al. showed that MCF-7 cells become resistant to tamoxifen or fulvestrant treatment when cultured with macrophages previously exposed to TNFα. This acquired resistance is dependent on ER phosphorylation at Ser118 induced by p42 MAPK and STAT3. This leads to the formation of an NF-κB/STAT3/phospho-ER complex that binds to the cyclin D1 gene promoter which, in turn, promotes cell proliferation independently of ER ligand status surpassing the efficacy of ER antagonists (230).

There is a strong body of evidence that shows that NF-κB modulates ER activity, thus modifying the response of ER-positive breast cancer cells to endocrine therapy. This is supported by the fact that NF-κB trans-represses ER functions that can proceed through protein-protein interaction or by recruitment of co-repressors. The lack of ER activation resulting from the administration of aromatase or ER inhibitors drives NF-κB-mediated tumor progression by releasing NF-κB from the inhibitory control of ER (231). Constitutive NF-κB activity and hyperactive p42/p44 MAPK are correlated with hormone resistance and emergence of ER-negative breast cancer cell populations (231). NF-κB activation also contributes to the aggressiveness of the tumor leading to the expression of several pro-survival genes, such as survivine and the multidrug transporter protein ABCG2 (206). The complexity of ER effects from liganded and unliganded ER activation and NF-κB activation through its canonical and non-canonical pathways, envision a broader network of interactions that would participate in hormone resistance which remains to be further explored.

In anti-estrogen-resistant LCC9 and MCF-7 cell lines, the small molecule inhibitor of the NF-κB pathway, parthenolide, was able to restore the sensitivity to fulvestrant *in vitro* (232). The LCC9 cells also exhibited a higher expression of p65 NF-κB than the parental cells. In line with these results, Zhou et al. using BT474 and MCF7/HER2 cell lines resistant to tamoxifen, demonstrated that NF-κB inhibition by parthenolide and bortezomib was able to overcome tamoxifen resistance. This effect was accompanied by the binding increase of the co-repressor NCOR1 to ER, which is associated with histone deacetylase activity and transcriptional repression of ER (233).

Clinical data support the *in vitro* findings. Several studies performed in ER breast cancer showed that the activation of NF-κB correlated with tamoxifen resistance, early relapse, a more aggressive tumor phenotype and a reduced overall survival (106, 234). It was demonstrated that the levels of NF-κB bound to DNA in breast cancer patients inversely correlate with ER expression in tissue samples (106). Taken as a whole, all these reports support the fact that crosstalk between ER and NF-κB pathways should be involved in the progression of ER-positive tumors, but do not take into account TNFα signaling. These findings propose a potential clinical use of NF-κB inhibitors to treat hormone-resistant breast cancer. Trinh et al. showed that the administration of bortezomib was able to delay disease progression in 22% of the patients with metastatic cancer resistant to endocrine therapy (235).

Although there are several reports showing TNFα participation in aromatase induction (described in the luminal breast cancer section), few studies have addressed its impact on aromatase inhibitors resistance. A report using genome wide expression profiling of 81 ER-positive breast carcinomas before and during treatment with an anastrozole in the neoadjuvant setting, identified an inflammatory signature that was correlated with poor response. The expressions of SLAMF8 and TNFα as well as lymphocytic infiltration were associated with lower effect on the antiproliferative response to the aromatase inhibitor. The data obtained in this study were validated in an independent cohort pointing to a clear involvement of TNFα in resistance to aromatase inhibitors.

### Tyrosine Kinases Inhibitors

Tyrosine kinases inhibitors (TKIs) are small molecules designed to compete with ATP at its binding site in the tyrosine kinase domain in the receptors to reduce phosphorylation. Their targets are proto-oncogene and oncogene products that have phospho-tyrosine kinase activities, thus inhibiting cancer cell proliferation. Several TKIs are used to treat breast cancer and are directed against EGFR (gefitinib, lapatinib, erlotinib), HER2 (lapatinib, neratinib), CDK4/6 (ribociclib, palbociclib, abemaciclib), and the PI3K/Akt/mTOR pathway (alpelisib, pictisilib, everolimus), among others [reviewed in (236)].

#### EGFR/HER2 Inhibitors

Lapatinib is a dual EGFR/HER2 tyrosine kinase inhibitor. It is used in metastatic breast cancer with capecitabine and with letozole in hormone positive HER2-positive breast cancer previously treated with trastuzumab (237), and as a second line of treatment in HER2-positive breast cancer after trastuzumab failure (238, 239). Wetterskog et al. identified NIK and IKKβ binding protein (NIBP) as a mediator of lapatinib resistance by studying HER2-amplified primary tumors and cell lines using a high throughput lapatinib small interfering RNA sensitivity screen, with a library targeting 369 genes recurrently amplified and overexpressed in HER2-positive breast cancer (240). It has been previously demonstrated that NIBP activated NF-κB signaling through IKKβ kinase (241). Therefore, the authors proposed the need of a simultaneous blockade of HER2 and NIBP that activates NF-κB by IKKβ and IKKβ activation, respectively, to overcome resistance to the TKI. Acquired resistance to lapatinib has been studied by several groups in HER2-positive breast cancer cell lines adapted to grow under lapatinib treatment. As constitutive activation of NF-κB of lapatinib-resistant cells is a common hallmark, inhibition of cell growth and apoptosis *in vitro* and *in vivo*, could be achieved by lapatinib administration together with an IKK inhibitor (242). Another work demonstrated that lapatinib resistance induced by NF-κB activation could be prevented by a sublethal concentration of an intracellular calcium chelator (212). In the case of hormone-positive HER2-positive breast cancer cell lines, the increased ER signaling and its transcriptional activity in response to lapatinib were described as a source of lapatinib resistance enhancing FOXO3a and caveolin-1 expression (212). On the other hand, lapatinib was also studied to treat TNBCs and was found to activate NF-κB via the Src family kinase and IκBα phosphorylation. This effect was not mediated by EGFR and only lapatinib, but no other EGFR inhibitor was able to synergize the anti-tumor activity of proteasome inhibitors *in vitro* and *in vivo* (243).

EGFR is frequently overexpressed in sporadic TNBC, and TKIs targeting EGFR are being used in clinical trials (213) [reviewed in (244)]. However, to the best of our knowledge, there are no reports showing the participation of TNFα or NF-κB in resistance to EGFR TKIs in breast cancer. In contrast, seminal findings by Bivona et al. have demonstrated in EGFR-mutant lung cancer cells that inhibition of NF-κB enhanced erlotinib-induced decrease in cell proliferation in erlotinib-sensitive and resistant cells (245). Then, the TRAF2-RIP1-IKK complex was identified as the signaling mediator of the NF-κB transcriptional activation induced by erlotinib (246). It has been recently demonstrated that EGFR inhibition by erlotinib in non-small cells lung cancer, independent of the EGFR mutation status, induced downregulation of miR-21, which in turn increased TNFα mRNA stability and its protein synthesis. TNFα then induced NF-κB activation and enhanced TNFα production itself in a feedforward loop. In the same experimental setting, blockade of TNFα enhanced EGFR TKIs antitumor effect in mutated EGFR tumors and overcame resistance to EGFR-targeted therapies. A similar mechanism of erlotinib resistance was found to take place in glioma cells (247).

#### PI3K Inhibitors

Alpelisib, a PI3K inhibitor, has been recently approved by the FDA for advanced or metastatic HER2-negative, hormone receptor positive with PIK3CA-mutation, that exhibits progression to endocrine therapy in breast cancer. However, up to today there have been no reports of resistance to alpeslisib linked to NF-κB or TNFα. However, *in vivo* findings using murine 4T1 TNBC cells co-cultured with macrophages, showed that pictisilib, another PI3K inhibitor, induced NF-κB activation in 4T1 cells and turned them resistant to this TKI. Addition of aspirin to the culture reinstated the apoptotic effect of pictisilib. Experiments performed *in vivo* proved that pictisilib administration induced the expression of macrophage-associated cytokines and chemokines, macrophage recruitment to the tumor bed, and poor antitumor effect. However, treatment with aspirin and pictisilib decreased macrophage infiltration, tumor growth and pulmonary metastasis (214), highlighting the importance of macrophage-induced NF-κB activation in tumor cells resistant to pictisilib. In line with these findings, TNFα derived from macrophages, turned melanoma cells resistant to MEK inhibition through activation of NF-κB (215).

#### CDK4/6 Inhibitors

Ribociclib, palbociclib and abemaciclib bind to CDK4 and 6 ATP pocket, which induces CDK4/6 inhibition. These drugs are used in metastatic hormone receptor positive, HER2-negative breast cancer together with aromatase inhibitors in first line or with fulvestrant as second line (216). The resistance mechanisms where NF-κB was involved were obtained from data in glioblastoma cell lines where it was found that abemaciclib treatment was ineffective to inhibit cell proliferation. This effect was produced by abemaciclib activation of NF-κB that, in turn, induced the production of hepatocyte growth factor, brain-derived neurotrophic factor, and nerve growth factor, activating c-Met and TrkA-B. Then, dual inhibition of c-Met/Trk and CDK4/6 was proposed for a clinical trial (248). In addition, prolonged exposure to pablociclib was reported to induce fibroblast senescence in an NF-κB dependent manner, and these cells were able to enhance melanoma growth in mice and recruitment of MDCS to the tumor bed.

### Poly-Adenosine Diphosphate (ADP) Ribose Polymerase (PARP) Inhibitors

The enzyme poly ADP ribose polymerase (PARP) repairs single strand breaks in DNA. Then, during DNA replication under treatment with PARP inhibitors, the single strand breaks result in double strand breaks when the DNA helix unwinds. Patients with hereditary breast and ovarian cancer syndrome have mutations in the homologous recombination repair enzymes BRCA1 and BRCA2, which are unable to repair these double strand breaks under PARP inhibitors treatment. The affected cells then enter apoptosis. PARP inhibitor-resistant breast and ovarian cancer cell lines harboring BRCA1 mutation were used to study gene expression by RNA sequencing (249). Pathway analysis showed that ~50% of the differentially expressed genes were NF-κB-regulated genes and that TNFα was the upstream regulator of several of them. It was also demonstrated that treatment of PARP inhibitor-resistant cells with bortezomib induced cell death to a greater extent than the parental cell line, confirming the involvement of the NF-κB pathway in the resistant phenotype. Bortezomib treatment increases PARP inhibitor sensitivity of the resistant cell lines, suggesting a possible therapeutic combination in the clinical setting (249).

### Trastuzumab

Trastuzumab, a humanized monoclonal antibody against HER2, has changed the poor outcome of patients with HER-positive breast cancer since its approval in 1998. Its therapeutic effect includes blockade of HER2 signaling through p42/p44 MAPK and PI3K/Akt pathways, inhibition of HER2 shedding, downregulation of HER2 availability, inhibition of tumor angiogenesis, and ADCC (217, 250–254). However, *de novo* and acquired resistance to trastuzumab reaches 27 and 42% in the adjuvant and neoadjuvant settings, respectively, hampering its clinical benefit (255, 256).

As mentioned before (HER2-positive section), NF-κB activation was found to be associated with ER-negative, HER2-positive breast cancer (257). Bortezomib treatment demonstrated to induce apoptosis and to act synergistically with trastuzumab in this breast cancer subtype (258, 259). In the case of HER2-positive ER-positive breast cancer cell lines resistant to trastuzumab, the NF-κB pathway showed to be constitutively activated and its blockade improved the tumor response to trastuzumab (260).

Our findings identified TNFα as an important player in trastuzumab resistance in HER2-positive breast and gastric cancer. We proved that TNFα overexpression turned trastuzumab-sensitive cells and tumors into resistant ones. On the other hand, *de novo* trastuzumab-resistant breast cancer cell lines exhibited greater TNFα levels than the sensitive ones (151). *In vivo* blockade of TNFα in mice bearing trastuzumab-resistant breast tumors inhibited tumor growth when trastuzumab was simultaneously administrated (151, 203). The antitumor effect was similar either when both tmTNF and sTNF were neutralized with etanercept, a fusion protein of TNFR2 and human IgG, or when only sTNF was blocked with INB03. Our study revealed that the mechanism underlying TNFα-induced trastuzumab resistance was mediated by MUC4 expression. MUC4 is masks the trastuzumab binding epitope on the HER2 molecule (261). Furthermore, MUC4 confers antiadhesive properties to tumor cells allowing their systemic dissemination. Our findings demonstrated that TNFα was able to upregulate MUC4 expression through NF-κB signaling pathway, impairing trastuzumab binding and ADCC. In addition, we proved that MUC4 expression in breast cancer specimens is an independent prognostic biomarker of poor response to adjuvant trastuzumab. These results suggest that MUC4 expression in HER2-positive breast cancer samples can be useful to select patients that can benefit from trastuzumab and TNFα-blocking therapies to avoid or overcome trastuzumab resistance (151). Our team also contributed to the characterization of MUC4 expression in different histological breast cancer subtypes. We identified that the invasive micropapillary carcinoma of the breast (IMPC), a low-incidence entity of breast cancer, was the one that had higher MUC4 expression in contrast to the infiltrating ductal carcinoma, the infiltrating lobular carcinoma or the mucinous carcinoma (262). In the HER2-positive breast cancers, IMPC was present in 18% of the samples in either its pure or mixed phenotype and was associated with hormone receptor expression. Despite the fact that HER2-positive/hormone receptor positive tumors have nowadays a prognosis similar to that of luminal B breast cancer (89), patients bearing IMPC exhibited a poor response to trastuzumab in the adjuvant setting (262).

### Immune Check-Point Inhibitors

In the past years, the impressive advances in immunotherapy irrupted into the cancer arena after the improvement of outcome in melanoma patients treated with the antibodies against the so-called immune checkpoints (263). After being triggered by an antigen, the physiological immune response goes through an effector phase that must be shut down, when the pathogen has been eliminated, to maintain homeostasis and prevent autoimmune symptoms or even autoimmune diseases. One of these self-control mechanisms relies on molecules that are expressed in the late effector phase of the immune response. CTLA-4 and PD-1 are molecules upregulated in activated T lymphocytes. They interact with CD80/CD86 or PD-L1 and PD-L2, respectively, which are present in dendritic cells, triggering negative signals in T lymphocytes. Tumor cells have the ability to express PD-L1 and/or PD-L2 mimicking cells from the host, thus impeding T lymphocytes attack. Therefore, the immunotherapeutic strategy that emerged is based on blocking CTL4/CD80/CD86 and PD-1/PD-L1 interaction with specific antibodies to avoid antitumor immune response shutdown (264). The FDA approved antibodies are: ipilimumab (anti-CTLA-4), pembrolizumab, nivolumab, cemiplimab-rwlc (anti-PD-1) and atezolimumab, avelumab, durvalumab (anti-PD-L1). They are used to treat bladder, cervical, colon, head and neck, liver, lung, renal, stomach cancers, and Hodgking lymphoma (265). The most promising results with immune checkpoint blockade (ICB) were obtained with nivolumab in refractory Hodgkin's lymphoma where the objective response rate was 87% (266). Thus, the reinvigoration of the antitumor immune response pursues the promotion of cancer cell cytotoxicity. The immunotherapy-based on atezolimumab has also recently reached FDA approval to treat TNBC, the breast cancer subtype with poorest survival, whose current treatment relies only on aggressive chemotherapy. In locally advanced or metastatic TNBC atezolizumab was found to be effective only in those tumors which express PD-L1 in their tumor-infiltrating lymphocytes (TILs) (267). However, the overall survival of these patients at 24 months was 50.7% in the atezolizumab combined with nab-paclitaxel group vs. 36.9% in the group treated with chemotherapy alone (267). In general, only a small number of the patients subjected to anti-immune checkpoint therapy experienced benefit. In addition, severe immune-related adverse events (irAEs) are frequently reported, and they stem from blocking the immune system control letting local and systemic autoimmune responses arise.

TNFα is an important player in the effectiveness of ICB. First, seminal findings by Lim et al. demonstrated the important participation of TNFα in PD-L1 biology (194). Using 4T1 cells and human TNBC cell lines, they demonstrated that TNFα induces COP9 signalosome 5 (C5N5), a protein that inhibits PD-L1 ubiquitination, through NF-κB activation. Then, PD-L1 protein increases its stability in tumor and dendritic cells, maintaining tumor immune evasion. In this experimental setting, the inhibition of NF-κB activation by curcumin was able to downregulate PD-L1 expression (194). Second, TNFα has been recognized as a mediator of ICB resistance in several cancer types. Preclinical studies performed in melanoma, lung and breast cancer show that TNFα induces expression of PD-L1. Treatment with anti-PD-1 allows TNFα-induced expression of T lymphocyte Immunoglobulin and TIM-3, another checkpoint molecule, in CD4+ and CD8+ TILs. In addition, TNFα also promotes death of CD8+ TILs (191). The above-mentioned effects were proved to be TNFR1-dependent, highlighting sTNFα's participation in immune surveillance evasion. The administration of anti-TNFα reduced CD8+ TILs cell death, TIM-3 and PD-L1 expression, decreasing T lymphocyte exhaustion upon anti-PD-1 therapy. In melanoma, anti-PD-1 therapy induced the regression of 20% of the tumors, while a synergistic antitumor effect was observed when anti-PD-1 was used in combination with anti-TNFα antibodies causing tumor regression of 75% (191).

As it was stated before, TNFR1 signaling can induce apoptosis or promote proliferation and survival depending on the adaptor proteins available in the cytoplasm. In the case of most cancer cells, TRAF2 is abundant, and the final outcome produced by TNFα is tumor growth. Recently, Vredevoogd et al., have described that TRAF2 induces resistance to T lymphocyte cytotoxicity in melanoma and lung cancer (175). Blockade of the TRAF2 pathway increased the susceptibility of these models to ICB. Consistently, patients with tumors harboring inactivating mutations in TRAF2 are more susceptible to T lymphocytes killing. On the other hand, it is known that hypoxia in solid tumors drives the formation of abnormal new vessels that provide an irregular blood flow and deficient drug delivery into the tumors. Concurrently, the endothelial cells of these vessels poorly express leucocyte adhesion molecules, impairing leukocytes extravasation. Fusion protein Cys–Asn–Gly–Arg–Cys–Gly–TNFα (NGR-TNFα), which targets the tumor vasculature delivering low amounts of TNFα, activates endothelial cells and allows CD8+ T lymphocytes infiltration (268). The simultaneous administration of NGR-TNFα with anti-PD-1 or anti-CTLA4 and with adoptive T lymphocyte therapy increased the effectiveness of each therapy alone in prostate and melanoma models (269). Finally, TNFα is induced by ICB and has been shown to be one of the important players in the irAEs. The irAEs, i.g. colitis, are managed by corticosteroid administration (270, 271), but refractory cases are treated with infliximab once ICB is suspended. Considering the aforementioned mechanisms of TNFα-induced ICB resistance, preclinical studies investigated the prophylactic effect of TNFα blockade both to impede irAEs development and to enhance ICB‘s efficacy. Melero and co-workers used etanercept prior to treatment with anti-CTLA4 and anti-PD-1 and found an improved anti-tumor efficacy of the ICB and also a decrease in colitis. These findings were achieved in tumor-transplanted mouse models of fibrosarcoma, melanoma, and colon cancer and in xenografts of colon cancer. This work verified an increase of CD8+ TILs, a decrease in T lymphocytes exhaustion and inactivation-induced cell death of T lymphocytes as described before (191). Based on this evidence, an ongoing clinical trial was launched in melanoma patients, combining nivolumab + Ipilimumab + the anti-TNFα certolizumab (272).

## Clinical Relevance of TNFα and its Blockade as Therapies in Cancer

Pioneer findings of Balkwill et al. demonstrated the usefulness of the treatment with anti-TNFα antibodies to prevent chemical-induced skin papilloma and inhibit growth of a transplantable breast cancer (273). These results opened up a new field of application of anti-TNFα drugs, so far devoted just to treat inflammatory and autoimmune diseases for more than 20 years.

The current anti-TNFα FDA approved therapies available are antibodies (infliximab, adalimumab, and golimumab), PEGylated Fab fragment (certolizumab pegol) and the fusion protein of TNFR2 with the Fc of the human IgG1 (etanercept). They all block both tmTNF and sTNF. As detailed above, active TNFα consists of a homotrimer that is continuously exchanging TNFα monomers. The dissociation rate is independent of TNFα concentration, while the association rate is not. The latter is very important in maintaining bioactivity at high concentrations, but not at low concentrations. The use of Förster resonance energy transfer (FRET) assay shed light on the difference of TNFα blockade with the above-mentioned inhibitors. In the case of adalimumab, infliximab, or etanercept treatment, the TNFα exchange between monomers and trimers is blocked but not by administration of certolizumab or golimumab. These findings might explain that under conditions of incomplete blocking, residual TNFα bioactivity can be found *in vitro* for adalimumab (274). The analysis of all the FDA approved drugs targeting TNFα revealed essentially no differences among them in the treatment of rheumatoid arthritis and spondyloarthritis (275). Regarding etanercept treatment, Liu et al. showed that certain polymorphisms in the TNFα gene promoter could predict a good response to TNFα-blocking therapies, especially for etanercept treatment in patients with spondyloarthritis (276). In this sense, another important contribution was made by Murdaca et al. that reported that the +489 TNFα polymorphism is associated with susceptibility and severity of psoriatic arthritis and this single nucleotide variant is responsible for a higher production of TNFα. Moreover, they informed that patients with +489 polymorphisms are associated with a better response to etanercept and adalimumab. On the contrary, in their cohort −238 and −408 polymorphisms do not influence the clinical outcome of patients with psoriatic arthritis which was related with the outcome of other auto-inflammatory immune diseases in other ethnicities (277). However, the application of these drugs in cancer will require a deeper study to determine which strategy would render the best outcome.

Due to TNFα‘s role in the immune system as a factor that causes tumor necrosis and controls infections, it was thought that the use of TNFα inhibitors could unleash cancer development and/or grant susceptibility to them. In this regard, there has been a good number of studies that addressed this issue in patients with inflammatory autoimmune diseases treated with different TNFα-blocking agents. No increased risk of developing malignancies was found in patients with rheumatoid arthritis and with immune-mediated diseases in general, psoriatic arthritis, ankylosing spondylitis and inflammatory bowel disease (278–283).

There is one study in patients with inflammatory bowel disease that previously had cancer, where the authors found that anti-TNFα treatment had a mild risk of incident cancer (284). These results are conflictive because it is well-known that patients who had a neoplastic disease in the past have an increased risk of developing new cancers, therefore the effect cannot be entirely attributed to anti-TNFα therapies. Current recommendations counterindicate the use of TNFα-blockers in patients with inflammatory bowel disease who had a cancer diagnosis in the last 5 years (285). On the other hand, there was another study of patients with rheumatoid arthritis and breast cancer that showed that TNFα-blockade did not increase recurrence of breast cancer with respect to patients treated otherwise (280).

In addition, two reports have described that anti-TNFα therapies cause an increased risk of non-melanoma skin cancers (279) and hematological malignancies in patients with rheumatoid arthritis. In the latter report it was also stated that rheumatoid arthritis patients have an increased risk of lymphomas due to the disease itself (286), which prevents us from speculating about the role of TNFα inhibitors.

The use of TNFα blocking agents for treating breast cancer has been poorly explored. In this regard there is only one phase II clinical trial that showed that etanercept is safe and well-tolerated in breast cancer patients, but that it did not produce objective disease responses, which could be due to the advanced stage of the patients (287). Taking all this into account, TNFα-blocking agents are a promising tool to treat breast cancer patients in combination with current cancer therapies.

Several findings support the fact that TNFR1 may be responsible for TNFα pro-tumorigenic actions [reviewed in (158, 288)]. If so, sTNFα neutralization would be an important path to explore. Indeed, Sobo-Vujanovic et al. and our own work demonstrated that sTNFα blockade prevents chemical induced skin carcinogenesis and overcomes trastuzumab resistance in HER2-positive breast cancer models, respectively (71, 151). The sTNFα blockade also preserves the cross talk between NK cells and dendritic cells that is mediated by tmTNFα, making less immunosuppressive targeting TNFα (14).

The facts exposed here postulate TNFα as an attractive target potentially useful to treat different breast cancer subtypes. These TNFα-blocking drugs should be studied in the clinical setting to potentiate current therapies and overcome treatment resistance thereby achieving a better outcome for the patients.

## Conclusion

TNFα has been proved to play a central role in initiation, promotion, and metastasis in most of cancers, in particular in breast cancer. It is also important at the onset of the immune response and exerts a cytotoxic effect against infected and in tumor cells. However, TNFα has a great variety of biological effects, from cell proliferation, inflammation, pro-survival activity, to cell death. These dramatically different outcomes are due to different TNFRs and the signaling adaptor proteins present in each cell, and to the different forms of TNFα: sTNFα and tmTNFα.

As cancer evolves after immunoedition acquires the ability to evade immunosurveillance. Along this process, tumor cells reduce their immunogenicity, and among other features, they become resistant to the cytotoxic activity of TNFα. The described adaptive mechanisms to avoid the lethal activity of TNFα include downregulation of TNFRs, increase of TNFR adaptor proteins which favors survival activity and glycosylation of TNFRs. In this scenario, TNFα induces survival and even cancer cell proliferation. Indeed, tumor cells themselves are able to produce TNFα and can increase its concentration upon therapy insults such as chemo/radiotherapy, targeted therapies and immunotherapy with ICB, to take advantage of NF-κB signaling to promote survival. In this way, it favors the emergence of therapy-resistant breast cancer cells directly linked to the recurrence of the disease.

The approval of ICB for the treatment of TNBC opens up a long sought new therapeutic possibility for this aggressive breast cancer subtype which is otherwise only handled with chemotherapy. However, irAEs frequently leads to ICB treatment withdrawal. As it is acknowledged that TNFα serum levels parallels the irAEs, the proposal of administration of TNFα blocking agents together with ICB will give chances to increase the number of patients able to complete the treatment. In addition, TNFα neutralization would also exert additional beneficial activities preventing T lymphocytes death and exhaustion.

On the other hand, TNFα also upregulates pro-metastatic factors, such as MMPs and MUC4, and enhances tumor dissemination. In particular, in HER2-positive breast cancer, MUC4 expression impairs trastuzumab efficacy by masking the binding epitope in the HER2 molecule and is an independent biomarker of poor survival in patients treated with adjuvant trastuzumab. All this evidence provides a proof-of-concept for proposing the blockade of TNFα as a promising therapy in breast cancer. Anti-TNFα therapies have been used for more than 20 years to treat autoimmune and inflammatory diseases. This fact envisions a shorter path to use TNFα-blocking agents together with the appropriate oncology treatments. Moreover, MUC4 expression in cancer specimens would help select the subgroup of HER2-positive breast cancer patients that benefit from the combination of anti-TNFα therapies with the standard treatments, as we previously demonstrated in HER2-positive breast cancer. This approach will contribute to precision medicine and will optimize the economic resources devoted to oncology.

## Author Contributions

All authors listed have made a substantial, direct and intellectual contribution to the work, and approved it for publication.

## Conflict of Interest

The authors declare that the research was conducted in the absence of any commercial or financial relationships that could be construed as a potential conflict of interest.
